# Effects of long-term preservation on amphibian body conditions: implications for historical morphological research

**DOI:** 10.7717/peerj.3805

**Published:** 2017-09-15

**Authors:** Guocheng Shu, Yuzhou Gong, Feng Xie, Nicholas C. Wu, Cheng Li

**Affiliations:** 1 Chengdu Institute of Biology, Chinese Academy of Sciences, Chengdu, Sichuan, China; 2 University of Chinese Academy of Sciences, Beijing, China; 3 School of Biological Sciences, University of Queensland, Brisbane, Queensland, Australia

**Keywords:** Effects, Preservation, Amphibian, Body length, Body mass, Morphological

## Abstract

Measurements of historical specimens are widely applied in studies of taxonomy, systematics, and ecology, but biologists often assume that the effects of preservative chemicals on the morphology of amphibian specimens are minimal in their analyses. We compared the body length and body mass of 182 samples of 13 live and preserved (up to 10 years) anuran species and found that the body length and body mass of preserved specimens significantly decreased by 6.1% and 24.8%, respectively, compared to those measurements of their live counterparts. The changes in body length and mass also exhibited highly significant variations between species. Similarly, there were significant differences in shrinkage of body length and body mass between sexes, where males showed greater shrinkage in body length and body mass compared to females. Preservation distorted the magnitude of the interspecific differences in body length observed in the fresh specimens. Overall, the reduction in body length or mass was greater in longer or heavier individuals. Due to the effects of preservation on amphibian morphology, we propose two parsimonious conversion equations to back-calculate the original body length and body mass of studied anurans for researchers working with historical data, since morphological data from preserved specimens may lead to incorrect biological interpretations when comparing to fresh specimens. Therefore, researchers should correct for errors due to preservation effects that may lead to the misinterpretation of results.

## Introduction

Common preservative chemicals, such as formalin and ethanol, are widely used in museum collections, especially for amphibian and reptile specimens (Simmons, 2002). However, due to the health risks to researchers from formalin (NRC, 1995) and the DNA degradation in formalin-fixed tissues (Wirgin et al., 1997), ethanol is more suitable for preserving amphibian specimens.

As biodiversity is rapidly declining around the world, museum specimens play an important role in biological research related to taxonomy (Arratia & Quezada-Romegialli, 2017), systematics (Huang et al., 2016), phylogeography (Godoy et al., 2004; Jaffe, Campbell-Staton & Losos, 2016), conservation biology (Parmesan, 1996), evolution (Losos & De Queiroz, 1997; Ochocińska & Taylor, 2003; Moen, 2006) and ecology (Irschick et al., 1997). Measurements of preserved specimens are often compared to those of live amphibians, especially when comparing historical records and evolutionary changes in morphology (Vervust, Van Dongen & Van Damme, 2009), but this method should be used with caution, as preservation may alter the overall appearance of animal specimens (Stuart, 1995). For example, the Australian green tree frog (*Litoria caerulea*) was originally described as the blue frog (*Rana caerulea*) because the preservation procedure removed the yellow pigments from the skin leaving only the blue and green pigments behind; the Latin name for blue is *caerulea* (Walls, 1995). Additionally, the body conditions (length and mass) of preserved specimens may also change over time (Fey & Hare, 2005; Melo et al., 2010). Thus, analyzing morphological data collected directly from historical specimens may generate misleading results.

Although a few researchers have examined the effects of preservation techniques on animal specimens, most herpetologists have not considered this problem. Scott Jr & Aquino-Shuster (1989) reported that frozen *Rana pipiens* specimens transferred to 40% isopropanol were softer with a duller overall appearance than non-frozen specimens, and they found that the snout-vent length (SVL) shrank 0–10% after one year, although the sample size was low (*n* = 7). Lee (1982) showed significant changes in 14 morphometric characters of *Rhinella marina* after six months of preservation in 70% ethanol; six morphological characters (e.g., SVL) increased, while the other eight decreased (e.g., axilla-groin length (AGL)). Surprisingly, repeated measurements after additional eight months showed that the changes in the 14 morphological traits (e.g., SVL) reversed compared to the first six months. Lee (1982) also found that preservation reduced the magnitude of the intersexual differences in fresh specimens and generated a new “sexual dimorphism”. In addition, not all characters were measurable with equal precision, and there was a highly significant correlation between precision and the inter-individual variation in characters. Another important confounding factor is inter-observer effects; Hayek, Heyer & Gascon (2001) examined researcher measurement error in frog morphometry in terms of both inter-observer effects on single measurements and intra-observer effects on repeated measurements of 14 characters of *Vanzolinius discodactylus* (Leptodactylidae) specimens. Based on statistical modeling, they argued that inter- and intra-observer differences in measurements may lead to different biological interpretations of results, and they also suggested that biologists should separately analyze data by sex and select the most appropriate statistical model for each data set. Meanwhile, large morphological characters (e.g., SVL or total length (TL)) have a lower intra-observer coefficient of variation and a greater precision than small characters (Yezerinac, Lougheed & Handford, 1992).

To date, most preservation studies have focused on one species with many morphological characteristics, except Deichmann, Boundy & Williamson (2009) who reported changes in the SVL of 14 anuran species in response to preservation. Their results revealed that 13 of these 14 species were significantly affected by the preservative with the SVL of all species decreasing by 0.31–5.62%. Across species, there was no evidence that smaller species shrank proportionately more or less than larger species. The authors also argued that most preservation-related changes occurred in the first several months after initial preservation, but they did not report on the long-term effects of preservation (>5 years).

All of the above researchers only examined the effects of relative short-term preservation on specimen morphology and neglected long-term effects, which are especially important in historical studies because many specimens are preserved for longer than has been previously reported. In this context, the objectives of this study were (1) to estimate the effects of long-term (10 years) ethanol preservation on amphibian (13 anuran species) body conditions, (2) to determine how differences in the change in body conditions across species, and (3) to provide conversion equations to correct for the body length and mass of preserved specimens and allow for a more accurate estimation of the body conditions of these historical specimens.

## Materials and Methods

### Ethics statement

The conducted research is in compliance with laws and ethical standards of the country. All animal procedures were approved by the Animal Care and Use Committee of the Chengdu Institute of Biology, Chinese Academy of Sciences (CIB2006062003). All field work with animals was conducted according to relevant national and international guide-lines. Chengdu Institute of Biology issued permit number CIB#2006-18 for field work.

### Data collection

A total of 182 specimens representing 13 anuran amphibian species, six families (Table 1) were collected in southwestern Sichuan in 2006 and stored at the Herpetological Museum of the Chengdu Institute of Biology, Chinese Academy of Sciences (CAS), where they were verified by amphibian experts. Sexual dimorphism was evident in a variety of the morphological traits of the frogs, such as body size, shape, and coloration; thus, male specimens were distinguished from female specimens according to their secondary sexual traits, including keratinized nuptial pads on the fingers, keratinized spines on the fingers and breast, cloacal dimorphism, a vocal sac, and the gonads.

**Table 1:** Information on the samples used in this study.

Family	Species	*N*	Location
Ranidae	*Amolops loloensis*	26	Zhaojue etc, Sichuan
	*Pelophylax nigromaculatus*	5	Miyi etc, Sichuan
	*Pseudorana weiningensis*	8	Zhaojue, Sichuan
	*Odorrana margaretae*	9	Dujiangyan etc, Sichuan
Dicroglossidae	*Nanorana pleskei*	10	Jiulong, Sichuan
	*Fejervarya multistriata*	5	Xichang, Sichuan
Megophryidae	*Scutiger glandulatus*	15	Kangding, Sichuan
	*Scutiger mammatus*	28	Jiulong etc, Sichuan
	*Oreolalax pingii*	14	Zhaojue, Sichuan
	*Atympanophrys shapingensis*	18	Zhaojue etc, Sichuan
Hylidae	*Hyla gongshanensis*	6	Xichang etc, Sichuan
Bufonidae	*Bufo gargarizans*	33	Xichang etc, Sichuan
Rhacophoridae	*Rhacophorus dugritei*	5	Mianning, Sichuan
6	13	182	

For consistency and to reduce measurement errors due to the position of the limbs and the position of the specimens fixed during preservation, we measured body mass (g) and SVL (mm) for amphibians. Hereafter, SVL is termed “body length”. The L_l_ and M_l_ of the specimens was measured during their initial capture while they were anesthetized. Body length was measured to the nearest 0.1 mm with dial calipers, and each individual was weighed using an electronic scale with a precision of 0.1 g. Absorbent paper was used to remove the excess fluids from the preserved specimens until there were no droplets forming when held. The specimens were weighed three times to account for fluctuations in readings. Live animals were euthanized via immersion in chloretone and water in compliance with the standards of the Animal Care and Use Committee of the Chengdu Institute of Biology, CAS.

All euthanized individuals were transferred to the Herpetological Museum, and the standard museum procedures were as follows: specimens were fixed in 10% formalin for 24 h, placed in standing water for an additional 24 h and subsequently transferred to 70% ethanol for long-term storage (Heyer et al., 1994; Simmons, 2002). In 2015, the same preserved specimens were measured again, including their L_p_ and M_p_, using the same dial calipers and scale. To determine the occurrence of inter-observer bias, two recorders measured three amphibian species independently, *Pseudorana weiningensis* (*n* = 8), *Amolops loloensis* (*n* = 26) and *Nanorana pleskei* (*n* = 10) and the results were compared.

### Statistical analysis

We examined the body length and body mass of 13 anuran species and compared changes between their live and preserved states. All data sets were tested for normality prior to other analyses, and a paired sampled *t*-test was applied to test for inter-observer variations in the measurements. Paired *t*-test and Wilcoxon signed-rank tests were used to analyze overall differences between L_l_ and L_p_, M_l_ and M_p_, and the analysis of covariance was preformed to compare interspecific differences in response to preservation. The paired *t*-test was also conducted to test the differences between L_l_ and L_p_, M_l_ and M_p_ in each sampled species. The independent sample *t*-test and Mann–Whitney U were carried out to analyze the differences in ACL, PCL, ACM, PCM, L_l_, L_p_, M_l_, and M_p_ between sexes and to test whether intersexual difference was changed during the preservation. Here, fifty females and fifty males were randomly sampled. Tamhane’s T2 (when the variances are unequal) were used for multiple comparisons in fresh specimens and preserved specimens respectively to ensure whether the interspecific differences were changed during the preservation. Linear correlation analysis was performed to test for relationships between the changes in body length or mass and the live length or mass of the amphibians. Finally, conversion equations were constructed to correct the body length and body mass measurements of preserved amphibian specimens through multiple-linear regression analysis, and the best models were determined by backward stepwise regression. A *t*-test was used to ascertain significant differences between the linear coefficient (*y*-intercept) and zero and the angular coefficient (slope) and one. All statistical tests were performed using SPSS (version 20.0; SPSS, Inc., Chicago, IL, USA), and differences were considered significant when *p* < 0.05. All of the results in the text are presented as the mean ± SD (standard deviation), except in the error bar charts, which show the mean ± SE (standard error).

## Results

### Reductions in body length and mass

There were no significant differences in both the body length and mass of the three species (*Pseudorana weiningensis*: *p* = 0.314, 0.351; *Amolops loloensis*: *p* = 0.607, 0.157; *Nanorana pleskei*: *p* = 0.399, 0.081; combined: *p* = 0.767, 0.087) measured by the two observers (Table  S1); thus, there is no inter-observer bias in our measurements. Overall, the paired *t*-test and Wilcoxon signed-rank test respectively revealed highly significant differences in body length (*t* = 21.911, *p* < 0.001) and body mass (*Z* =  − 13.789, *p* < 0.001) between the live and preserved specimens, where the body length and body mass of the preserved specimens significantly decreased relative to those of the fresh specimens. The changes in body length and mass also exhibited highly significant variations between species (body length: absolute change, *F*_12,169_ = 10.197, *p* < 0.001; percent change, *F*_12,169_ = 4.873, *p* < 0.001; body mass: absolute change, *F*_12,169_ = 10.131, *p* < 0.001; percent change, *F*_12,169_ = 21.649, *p* < 0.001).

**Figure 1:**
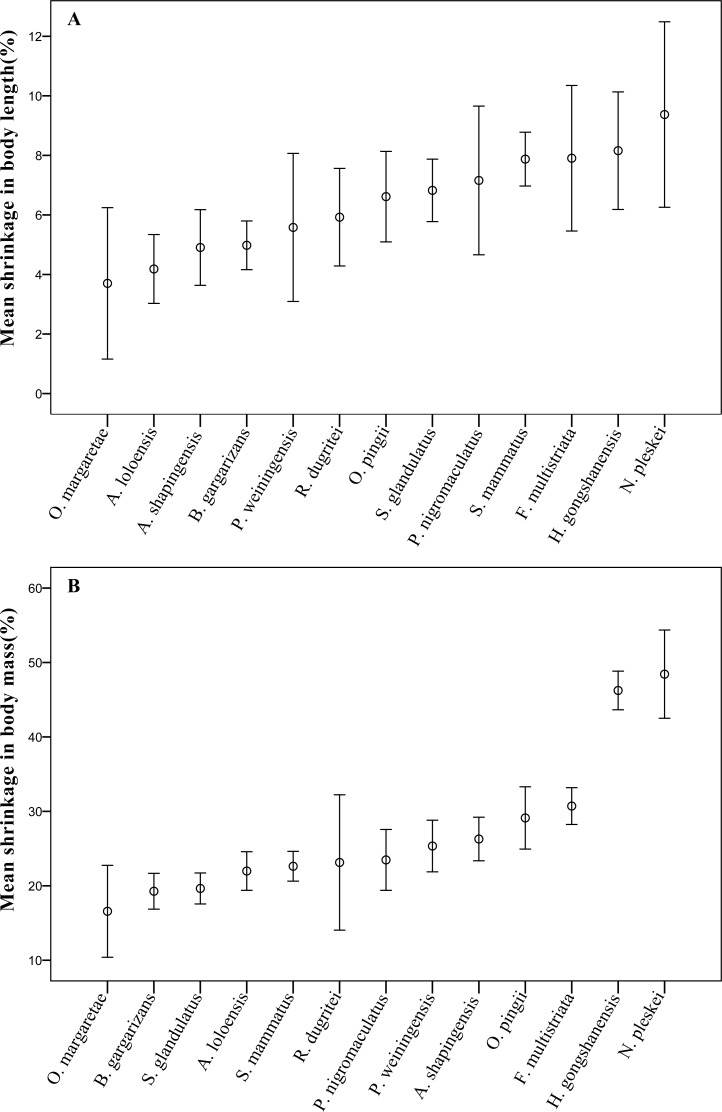
The mean shrinkage in (A) body length (%), and (B) body mass (g) across 13 anuran amphibian species over the 10-year preservation period. Error bars indicate standard errors (SE).

Furthermore, the results showed that the mean body length or mass of each sampled species were significantly (*p* < 0.05) different between pre-preservation and post-preservation (Tables S2 and S3). The body lengths of six species were markedly significantly (*p* < 0.01) reduced during preservation; and those of eight species were highly significantly (*p* < 0.001) reduced. The body mass results were similarly observed (Table  S3). The mean reductions in body length and body mass varied between species by 6.06 ± 3.17% and 24.82 ± 10.08%, respectively. Both the body length and mass of *Odorrana margaretae* shrank the least with length shrinking by 3.70 ± 3.81% and mass by 16.57 ± 9.26%, and the body length of *Nanorana pleskei* shrank the most by 9.37 ± 4.92%. Surprisingly, *Nanorana pleskei* shrank by 48.43 ± 9.38% on average, roughly half its entire body mass (Fig. 1).

The effects of preservation (ACL and ACM) on body length and body mass did not differ significantly between sexes, but the percent change in body conditions (PCL and PCM) after preservation was significant greater in males than females (PCL: *t* = 2.269, *P* = 0.025; PCM: *t* = 3.386, *P* = 0.001; Table 2).

**Table 2:** Differences in measurements by sex (male and female) of all 13 amphibians in this study.

Trait	Male (*N* = 50)	Female (*N* = 50)	Mean difference	*t*/Z	*P*-value
ACL (mm)	4.31 ± 2.22	4.05 ± 2.33	−0.26 ± 0.46	0.562	0.576
PCL (%)	6.70 ± 2.66	5.39 ± 3.09	−1.31 ± 0.58	2.269	0.025
ACM (g)	6.64 ± 5.01	7.82 ± 4.80	1.18 ± 0.98	−1.462	0.144
PCM (%)	25.96 ± 9.41	19.65 ± 9.24	−6.31 ± 1.86	3.386	0.001
L_l_	63.32 ± 17.70	76.65 ± 13.69	13.33 ± 3.17	−4.211	<0.001
L_p_	59.01 ± 16.17	72.59 ± 13.62	13.58 ± 2.99	−4.544	<0.001
M_l_	28.42 ± 20.15	42.73 ± 21.48	14.31 ± 4.17	−3.435	0.001
M_p_	21.78 ± 15.67	34.91 ± 18.88	13.13 ± 3.47	−3.783	<0.001

### Distortion of interspecific differences with long-term preservation

We conducted multiple comparisons of the body characteristics of species when fresh and after preservation respectively to test whether the interspecific differences were changed during the preservation. In total, there were 156 species pairs referring to random two species from 13 species (78 species counterparts, and each counterpart had two indicators, i.e., length and mass). Multiple comparisons indicated that long-term preservation significantly distorted the interspecific differences in 13 of the 156 species pairs, and six preserved pairs exhibited greater interspecific differences than detected in their live counterparts. However, the seven pairs presented interspecific differences of lower magnitude than in the live specimens (Table S4). Furthermore, when comparing body length, the significant differences in six pairs disappeared after preservation; a marginally significant difference (*p* < 0.05) in one pair increased to a markedly significant difference (*p* < 0.01); and differences in three pairs increased from being markedly significant to highly significant (*p* < 0.001). In body mass, by contrast, a significant difference occurred in one pair after preservation; the difference in one pair disappeared; and the difference increased to marked significance from marginal significance in one pair and to high significance from marked significance in another. The magnitudes of these changes in interspecific differences were shown in Table S4 .

### Correlation analysis and correction equations

Because of the great reduction in body length, we determined whether L_p_, ACL, and PCL were correlated with L_l_ (Table 3) in all individuals, and the relationships between M_l_ and M_p_ and between shrinkage (ACM and PCM) and M_l_ were also determined. The results indicated that there was a strong correlation between L_l_ and L_p_ (*R* = 0.995, *p* < 0.001) and between L_l_ and ACL (*R* = 0.500, *p* < 0.001) in 13 anurans as well as between M_l_ and M_p_ (*R* = 0.990, *p* < 0.001) and between ACM and M_l_ (*R* = 0.843, *p* < 0.001). In other words, longer individuals showed a greater reduction (absolute change) in body length than shorter individuals, and the body mass of heavier individuals was reduced more (absolute change) than that of lighter individuals. However, PCL was not associated with L_l_ and PCM was negatively significantly correlated with M_l_ (*R* =  − 0.447, *p* < 0.001) in sampled species. Thus, the shrinkages in body length and body mass were not parallel.

**Table 3:** Correlation coefficient for the relationships between L_**p**_ and L_**l**_; shrinkage in body length (ACL and PCL) and L_**l**_; M_**p**_ and M_**l**_; shrinkage in mass (ACM and PCM) and M_**l**_; ACL and ACM; PCL and PCM.

Trait	Correlation coefficient	*P*-value
L_p_ and L_l_	0.995	<0.001
ACL and L_l_	0.500	<0.001
PCL and L_l_	−0.129	0.084
M_p_ and M_l_	0.990	<0.001
ACM and M_l_	0.843	<0.001
PCM and M_l_	−0.447	<0.001
ACL and ACM	0.545	<0.001
PCL and PCM	0.299	<0.001

**Figure 2:**
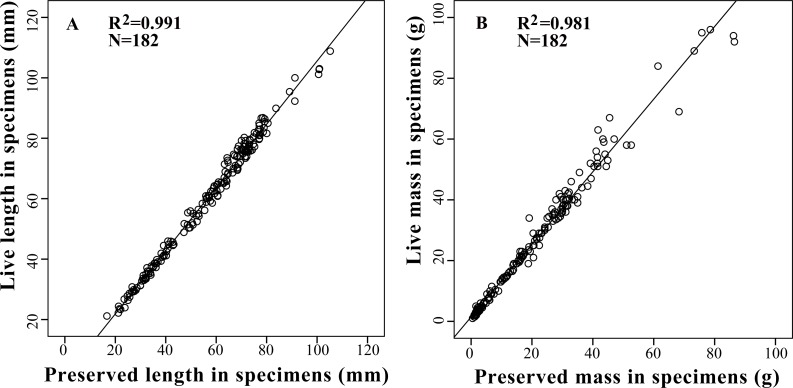
Relationships between live body length (A) and body mass (B) with preserved body length (A) and body mass (B) in 13 tailless amphibian species. Regression line represents the slope of correlation between live and preserved variables with a *R*^2^ of 0.99 for body length and 0.98 for body mass.

Based on the results of the correlation analysis, relative conserved correction equations were constructed to estimate the live length and mass of amphibians from preserved specimens which were listed in the study via multiple-linear regression analysis (Fig. 2). The most optimal models were presented in Table 4.

**Table 4:** Correction equations to estimate the fresh body length and body mass for all 13 amphibian specimens in this study.

Regression equation	*N*	*R*^2^	*t*-test slope = 1	*P*-value	*t*-test *y*-intercept = 0	*P*-value
L_l_ = 1.046 L_p_ + 0.934	182	0.991	2.070	<0.001	137.754	0.040
M_l_ = 1.195 M_p_ + 1.438	182	0.981	4.340	<0.001	96.506	<0.001

## Discussion

Long-term preservation caused pronounced reductions in body measurements in 13 amphibian species. For all 13 species studied, there were significant or highly significant differences in body length and mass between live specimens and preserved specimens. Interestingly, the sample sizes of the species in which there were no highly significant pre-preservation and post-preservation differences, such as *R. dugritei, P. nigromaculatus, F.  multistriata* and *H. gongshanensis*, were the less numerous in the study, which suggested that the number of animals sampled needs to be took into account, as observed by Deichmann, Boundy & Williamson (2009). The differences in shrinkage between species ranged from 3.70% to 9.37% in body length and 16.57% to 48.43% in body mass, especially the shrinkage in body length was not fully consistent with other studies, and the other studies rarely reported the shrinkage in body mass. For example, Lee (1982) found that the SVL of *Rhinella marina* increased by 1.68% after 14 months of preservation in 70% ethanol, and Deichmann, Boundy & Williamson (2009) reported that preservation in 70% ethanol significantly influenced the SVL of 13 of 14 species that had been preserved for 5 years or less, with 0.31–5.62% SVL shrinkage. So, the findings of Deichmann, Boundy & Williamson (2009) were not fully consistent with our results; our long-term study showed greater shrinkage in body length, which suggests that the duration of preservation may drastically influence body characteristics and lead to variations in shrinkage. However, the rate of shrinkage over time can only be determined by measuring specimens over a set period of time. Thus, a further study is necessary. Given the specimens in our study exhibited great changes in body length and mass over longer preservation periods, so uninformed researchers focusing on taxonomy, phylogeny, ecology and evolutionary morphology could potentially get misleading results if they use direct measurements of preserved specimens to infer the characteristics of their live counterparts.

Preservation impacted interspecific differences, which varied with preservation time, as well as differences related to sex and body size or shape. Because ethanol penetrates and dehydrates tissues (Sturgess & Nicola, 1975), the shrinkage of specimens mainly resulted from the loss of water during the process of preservation. Male amphibians shrank more in both body length and mass compared to females. Typically, female amphibians are larger than males, although *S. mammatus* was an exception in this study (Fairbairn, Blanckenhorn & Székely, 2007; Fei, Ye & Jiang, 2012). Therefore, body size influences the rate of ethanol dehydration due to the scaling exponent of the surface area to volume ratio (Klein et al., 2016), where smaller animals have a disproportionally larger surface area to volume ratio (exponent 0.68). The 13 amphibian species also differ in body shape; thus, the differences in shrinkage in this study can be explained by differences in body size or shape between sexes and species.

The shrinkages presented high variations among 13 species, which might indirectly reflect phylogeny status of these species since they come from different genera or families. Also, arboreal species tend to have the high resistance to evaporative water loss, compared to aquatic species, which tend to have little or no skin resistance, and terrestrial species tend to have resistance between those of arboreal and aquatic frogs (Young et al., 2005), which may explain the differences in the rate of shrinkage in this study. For example, *F. multistriata* shrank more than most of sampled species in this study, which is presumably an aquatic taxon (IUCN, 2016). However, all of the other sampled species in this study belonged to the semi-aquatic group, whose habitats are relatively drier (IUCN, 2016). Schmid (1965) also reported that nine amphibian species exhibited marked variation in their habitat preferences in terms of the availability of water. Therefore, the rate of shrinkage may also relate to habitat selection by the different species.

Various skin characteristics can also change the rate of water loss, including skin texture, thickness, and the presence of cutaneous glands (Toledo & Jared, 1993; Torri et al., 2014). Schmid & Barden (1965) found an inverse correlation between the permeability to water and the lipid content of skin. The lipid content of the skin might influence the rate of water loss during long-term preservation, resulting in interspecific differences in shrinkage. The SVL of green iguanas (*Iguana iguana*) preserved in 70% ethanol shrank between 1–7% over a two-month period (Vervust, Van Dongen & Van Damme, 2009), and Reed (2001) similarly reported that the SVL of snakes (41 species) decreased by 6–7% in 70% ethanol (16 years, 4 years and <1 year), while there was little reduction in mass (ranged from 0.772 to 1.267 grams). This suggests that although the longest preservation time studied by Reed (2001) was longer than ours, using the same type and concentration of preservatives, the maximum SVL shrinkage in snakes was less than in amphibians. Thus, skin structure plays an important role in the rate of water loss during long-term preservation, where animals with a thicker epidermis such as snakes may show less shrinkage compared to amphibians (Vitt & Caldwell, 2013). Lee (1982) speculated that intersexual differences in shrinkage might also be related to the reproductive condition of females, which can carry a rich complement of eggs, but this remains to be tested. The variations in body shrinkage observed in this study could potentially be explained by complex interactions among the factors mentioned above (Arratia & Quezada-Romegialli, 2017).

Based on this research, we conclude that long-term storage greatly deforms the body characteristics of tailless amphibians, which will confound results if historical specimens are directly measured. For example, we found that preservation changed the magnitude of the interspecific differences in body conditions detected in fresh specimens, which was similar to the results of Lee (1982), and correlation analysis indicated that L_p_ and ACL were significantly correlated with L_l_. Overall, the reduction in body length or mass was greater in longer and heavier anuran individuals. However, the percent body length shrinkage values were not associated with the live length in sampled species, which implies that differences among and within species may be obliterated during long-term preservation, or preservation might exaggerate similarities or differences between large and small specimens (Lee, 1982; Hayek, Heyer & Gascon, 2001). Consequently, preserved specimens are unlikely to accurately reflect the morphologies of live specimens, which may lead to different biological interpretations and result in false conclusions.

In summary, long-term preservation significantly influences the body characteristics of amphibian specimens and can distort interspecific and inter-individual differences. Therefore, researchers need to consider the influence of preservation on morphology when interpreting the results of ecological and taxonomic studies involving historical specimens, especially if the sampled specimens are being compared with fresh individuals. Fortunately, the length and mass of living individuals can be predicted from preserved specimens using conversion equations, as has been done with medusae (Thibault-Botha & Bowen, 2004), lizards (Vervust, Van Dongen & Van Damme, 2009), insects (Lee, Kodama & Horiguchi, 2012), and especially fish (Shields & Carlson, 1996; Porter, Brown & Bailey, 2001; Fey & Hare, 2005; Thorstad et al., 2007; Melo et al., 2010). Therefore, we suggest two parsimonious conversion equations to estimate the fresh length and mass from tailless amphibian specimens (species listed in this study) preserved in 70% ethanol over the long term to improve the reliability of morphological data from historical specimens. Museums are valuable resources for ancient and rare specimens; thus, obtaining accurate measurements from preserved specimens will allow for accurate interpretations of biological change over the short or long terms.

##  Supplemental Information

10.7717/peerj.3805/supp-1 Table S1 Size range of frogs measured and the difference in measurements between researchers

10.7717/peerj.3805/supp-2 Table S2 Range and mean values of live and preserved body length

10.7717/peerj.3805/supp-3 Table S3 Range and mean values of live and preserved body mass

10.7717/peerj.3805/supp-4 Table S4 Multiple comparisons between fresh specimens and preserved specimens

10.7717/peerj.3805/supp-5 Data S1 The raw data in this study

10.7717/peerj.3805/supp-6 Data S2 The raw data for test inter-observer bias

## Abbreviations

L_**l**_: live body length
L_**p**_: preserved body length
M_**l**_: live body mass
M_**p**_: preserved body mass
ACL: the absolute change in body length
PCL: the percent change in body length
ACM: the absolute change in body mass
PCM: the percent change in body mass
SVL: snout-vent length

## Additional Information and Declarations

### Field Study Permissions

The following information was supplied relating to field study approvals (i.e., approving body and any reference numbers): Chengdu Institute of Biology issued permit number CIB#2006-18 for field work.

### Data Availability

The following information was supplied regarding data availability: The raw data has been provided as Supplemental Files.
