# Supplementary material for: Effects of long-term preservation on amphibian body conditions: implications for historical morphological research
Source: PeerJ. 2017 Sep 15;5:e3805. doi: 10.7717/peerj.3805 (PMC5602676; doi:10.7717/peerj.3805)
Supplement: Table S1 [file peerj-05-3805-s001.docx]

Table S1. Size range of frogs measured and the difference in measurements between researchers (CL-GCS) (Paired-t Test). Data was presented as mean±SD.

| Species | N | L_p_  Mean (mm)  CL/GCS | M_p_  Mean (g)  CL/GCS | L_p_ Mean difference  (mm) | M_p_ Mean  Difference  (g) | L_p_  Mean% difference | M_p_  Mean% difference | *t* | P-value  For L_p_ | *t* | P-value  For M_p_ |
| --- | --- | --- | --- | --- | --- | --- | --- | --- | --- | --- | --- |
| *Pseudorana weiningensis* | 8 | 25.68±5.14  25.64±5.20 | 1.72±0.84  1.71±0.85 | 0.04±0.11 | 0.01±0.04 | 0.20±0.45 | 1.04±2.76 | 1.085 | 0.314 | 1.000 | 0.351 |
| *Amolops loloensis* | 26 | 55.45±12.69  55.54±12.94 | 15.85±10.47  15.92±10.35 | -0.09±0.91 | -0.07±0.26 | 0.05±1.58 | 1.34±1.93 | -0.521 | 0.607 | 1.459 | 0.157 |
| *Nanorana pleskei* | 10 | 28.42± 5.52  28.35±5.60 | 1.55±0.60  1.52±0.59 | 0.07±0.25 | 0.03±0.05 | 0.30±0.76 | 2.11±3.59 | 0.886 | 0.399 | 1.964 | 0.081 |
| Total | 44 | 43.89±17.39  43.92±17.56 | 10.07±10.63  10.02±10.68 | -0.03±0.71 | 0.05±0.20 | 0.06±1.30 | 1.48±2.63 | -0.298 | 0.767 | 1.749 | 0.087 |
