# Supplementary material for: Effects of long-term preservation on amphibian body conditions: implications for historical morphological research
Source: PeerJ. 2017 Sep 15;5:e3805. doi: 10.7717/peerj.3805 (PMC5602676; doi:10.7717/peerj.3805)
Supplement: Table S2 [file peerj-05-3805-s002.docx]

Table S2. Range and mean (± SD) values of L_l_ (live length) and L_p_ (preserved length), and the change in body length (mm) after being preserved in ethanol preservative (Paired-t Test).

| Species | N | L_l_  Range (mm) | L_p_  Range (mm) | Mean  L_l_ (mm) | Mean  L_p_ (mm) | Mean  Difference (mm) | Shrinkage  (%) | *t* | *P*-value |
| --- | --- | --- | --- | --- | --- | --- | --- | --- | --- |
| *O. margaretae* | 9 | 35.2-108.8 | 33.2-105.2 | 79.0±27.9 | 76.6±28.4 | 2.4±2.0 | 3.70 ±3.81 | 3.599 | 0.007 |
| *A. loloensis* | 26 | 33.9-76.1 | 32.0-74.2 | 57.6±12.6 | 55.2±12.5 | 2.4±1.8 | 4.18±2.95 | 6.572 | ＜0.001 |
| *B. gargarizans* | 33 | 44.5-95.4 | 39.2-89.1 | 69.5±11.8 | 66.0±11.0 | 3.5±1.7 | 4.97 ±2.35 | 11.620 | ＜0.001 |
| *A. shapingensis* | 18 | 32.8-81.9 | 30.5-80.0 | 60.9±16.7 | 57.8±15.5 | 3.1±2.2 | 4.90±2.69 | 5.930 | ＜0.001 |
| *P. weiningensis* | 8 | 22.2-33.5 | 21.2-32.2 | 27.2±5.1 | 25.7±5.1 | 1.5±0.8 | 5.58±3.52 | 4.994 | 0.002 |
| *R. dugritei* | 5 | 39.0-61.8 | 36.9-59.4 | 45.7±9.1 | 43.1±9.2 | 2.6±0.6 | 5.92 ±1.83 | 9.371 | 0.001 |
| *O. pingii* | 14 | 26.4-45.9 | 25.0-42.1 | 33.9±6.2 | 31.7±6.1 | 2.2±1.0 | 6.61 ±2.84 | 8.411 | ＜0.001 |
| *S. glandulatus* | 15 | 51.7-78.3 | 47.3-75.2 | 70.6±8.1 | 65.8±7.7 | 4.8±1.6 | 6.83 ±2.03 | 11.543 | ＜0.001 |
| *P. nigromaculatus* | 5 | 65.5-83.5 | 60.9-78.9 | 77.0±6.9 | 71.5±6.6 | 5.5±2.3 | 7.16±2.79 | 5.331 | 0.006 |
| *F. multistriata* | 5 | 37.1-41.3 | 32.6-39.3 | 38.7±1.6 | 35.6±2.4 | 3.0±1.0 | 7.90±2.73 | 7.100 | 0.002 |
| *S. mammatus* | 28 | 63.5-100.0 | 58.9-91.2 | 78.0±7.9 | 71.9±7.3 | 6.2±2.0 | 7.87 ±2.39 | 16.576 | ＜0.001 |
| *H. gongshanensis* | 6 | 28.7-45.9 | 25.6-40.9 | 34.2±6.6 | 31.4±5.5 | 2.9±1.3 | 8.15 ±2.42 | 9.371 | 0.003 |
| *N. pleskei* | 10 | 21.2-37.7 | 16.7-35.00 | 32.1±5.3 | 29.2±5.8 | 2.8±1.0 | 9.37±4.92 | 9.365 | ＜0.001 |
