# Supplementary material for: Effects of long-term preservation on amphibian body conditions: implications for historical morphological research
Source: PeerJ. 2017 Sep 15;5:e3805. doi: 10.7717/peerj.3805 (PMC5602676; doi:10.7717/peerj.3805)
Supplement: Table S3 [file peerj-05-3805-s003.docx]

Table S3. Range and mean (± SD) values of M_l_ (live mass) and M_p_ (preserved mass), and the change in mass (g) after being preserved in ethanol preservative (Paired-t Test).

| Species | N | M_l_  Rrange (g) | M_p_  Rrange (g) | Mean  M_l_ (g) | Mean  M_p_ (g) | Mean  Difference (g) | Shrinkage  (%) | *t* | *P*-value |
| --- | --- | --- | --- | --- | --- | --- | --- | --- | --- |
| *O. margaretae* | 9 | 3.5-96.0 | 2.7-86.5 | 54.3±40.9 | 47.7±37.2 | 6.6±6.1 | 16.57±9.26 | 3.244 | 0.012 |
| *B. gargarizans* | 33 | 8.0-84.0 | 5.9-61.4 | 33.2±17.00 | 26.7±13.1 | 6.5±4.8 | 19.26±6.92 | 7.798 | ＜0.001 |
| *S. glandulatus* | 15 | 10.0-44.0 | 9.0-36.4 | 31.8±9.9 | 25.4±8.0 | 6.3±2.3 | 19.64±4.03 | 10.739 | ＜0.001 |
| *A. loloensis* | 26 | 3.5-41.0 | 2.4-35.0 | 20.1±12.0 | 15.9±9.9 | 4.2±2.9 | 21.98±6.61 | 7.388 | ＜0.001 |
| *R. dugritei* | 5 | 6.0-25.0 | 4.9-20.6 | 11.4±7.8 | 8.8±6.6 | 2.6±1.6 | 23.14±10.17 | 3.655 | 0.022 |
| *S. mammatus* | 28 | 21.0-95.0 | 16.1-75.8 | 43.4±16.2 | 33.6±12.8 | 9.8±4.5 | 22.63±5.30 | 11.677 | ＜0.001 |
| *P. nigromaculatus* | 5 | 24.5-59.0 | 19.0-43.6 | 44.7±13.1 | 34.1±9.9 | 10.6±4.0 | 23.47±4.57 | 5.917 | 0.004 |
| *A. shapingensis* | 18 | 3.0-52.0 | 1.9-41.5 | 21.8±14.8 | 16.5±11.5 | 5.3±3.6 | 26.28±6.20 | 6.366 | ＜0.001 |
| *P. weiningensis* | 8 | 1.5-4.0 | 1.0-2.9 | 2.3±1.1 | 1.7±0.8 | 0.6±0.3 | 25.34±4.90 | 5.298 | 0.001 |
| *O. pingii* | 14 | 2.0-7.5 | 1.4-5.7 | 3.7±1.8 | 2.6±1.2 | 1.1±0.7 | 29.11±7.83 | 5.832 | ＜0.001 |
| *F. multistriata* | 5 | 4.0-6.0 | 2.6-4.1 | 4.9±0.7 | 3.4±0.5 | 1.5±0.2 | 30.71±2.76 | 17.516 | ＜0.001 |
| *H. gongshanensis* | 6 | 2.5-9.0 | 1.3-5.2 | 4.3±2.4 | 2.4±1.4 | 1.9±1.0 | 46.24±3.18 | 4.972 | 0.004 |
| *N. pleskei* | 10 | 1.0-5.0 | 0.5-2.4 | 3.2±1.4 | 1.6±0.6 | 1.6±0.9 | 48.43±9.38 | 5.642 | ＜0.001 |
