# Supplementary material for: Effects of long-term preservation on amphibian body conditions: implications for historical morphological research
Source: PeerJ. 2017 Sep 15;5:e3805. doi: 10.7717/peerj.3805 (PMC5602676; doi:10.7717/peerj.3805)
Supplement: Table S4 [file peerj-05-3805-s004.docx]

Table S4. Multiple comparisons of fresh specimens and preserved specimens respectively (Tamhane’s T2).

| Pairs | *P*-value | | | |
| --- | --- | --- | --- | --- |
|  | Body length | | Body mass | |
|  | Difference in L_l_ | Difference in L_p_ | Difference in M_l_ | Difference in M_p_ |
| *A. loloensis* VS. *F. multistriata* | ＜0.001*** | ＜0.001*** | ＜0.001*** | ＜0.001*** |
| *A. loloensis* VS. *S. glandulatus* | 0.024* | **0.149** | 0.161 | 0.159 |
| *A. loloensis* VS. *N. pleskei* | ＜0.001*** | ＜0.001*** | ＜0.001*** | ＜0.001*** |
| *A. loloensis* VS. *P. weiningensis* | ＜0.001*** | ＜0.001*** | ＜0.001*** | ＜0.001*** |
| *A. loloensis* VS. *A. shapingensis* | 1.000 | 1.000 | 1.000 | 1.000 |
| *A. loloensis* VS. *O. margaretae* | 0.993 | 0.995 | 0.968 | 0.955 |
| *A. loloensis* VS. *H. gongshanensis* | 0.001** | ＜0.001*** | ＜0.001*** | ＜0.001*** |
| *A. loloensis* VS. *B.gargarizans* | 0.047* | **0.093** | 0.084 | 0.060 |
| *A. loloensis* VS. *P. nigromaculatus* | 0.056 | 0.126 | 0.594 | 0.613 |
| *A. loloensis* VS. *S. mammatus* | ＜0.001*** | ＜0.001*** | ＜0.001*** | ＜0.001*** |
| *A. loloensis* VS. *O. pingii* | ＜0.001*** | ＜0.001*** | ＜0.001*** | ＜0.001*** |
| *A. loloensis* VS. *R. dugritei* | 0.977 | 0.973 | 0.999 | 1.000 |
| *F. multistriata* VS. *S. glandulatus* | ＜0.001*** | ＜0.001*** | ＜0.001*** | ＜0.001*** |
| *F. multistriata* VS. *N. pleskei* | 0.287 | 0.625 | 0.523 | 0.025* |
| *F. multistriata* VS. *P. weiningensis* | 0.021* | **0.064** | 0.038* | **0.106** |
| *F. multistriata* VS. *A. shapingensis* | 0.003** | 0.001** | 0.014* | 0.014* |
| *F. multistriata* VS. *O. margaretae* | 0.201 | 0.201 | 0.461 | 0.483 |
| *F. multistriata* VS. *H. gongshanensis* | 1.000 | 1.000 | 1.000 | 1.000 |
| *F. multistriata* VS. *B.gargarizans* | ＜0.001*** | ＜0.001*** | ＜0.001*** | ＜0.001*** |
| *F. multistriata* VS. *P. nigromaculatus* | 0.014* | 0.007** | 0.196 | 0.184 |
| *F. multistriata* VS. *S. mammatus* | ＜0.001*** | ＜0.001*** | ＜0.001*** | ＜0.001*** |
| *F. multistriata* VS. *O. pingii* | 0.796 | 0.996 | 0.997 | 0.998 |
| *F. multistriata* VS. *R. dugritei* | 1.000 | 1.000 | 1.000 | 1.000 |
| *S. glandulatus* VS. *N. pleskei* | ＜0.001*** | ＜0.001*** | ＜0.001*** | ＜0.001*** |
| *S. glandulatus* VS. *P. weiningensis* | ＜0.001*** | ＜0.001*** | ＜0.001*** | ＜0.001*** |
| *S. glandulatus* VS. *A. shapingensis* | 0.973 | 0.998 | 0.931 | 0.710 |
| *S. glandulatus* VS. *O. margaretae* | 1.000 | 1.000 | 1.000 | 1.000 |
| *S. glandulatus* VS. *H. gongshanensis* | ＜0.001*** | ＜0.001*** | ＜0.001*** | ＜0.001*** |
| *S. glandulatus* VS. *B.gargarizans* | 1.000 | 1.000 | 1.000 | 1.000 |
| *S. glandulatus* VS. *P. nigromaculatus* | 1.000 | 1.000 | 1.000 | 1.000 |
| *S. glandulatus* VS. *S. mammatus* | 0.495 | 0.814 | 0.404 | 0.736 |
| *S. glandulatus* VS. *O. pingii* | ＜0.001*** | ＜0.001*** | ＜0.001*** | ＜0.001*** |
| *S. glandulatus* VS. *R. dugritei* | 0.120 | 0.213 | 0.100 | 0.134 |
| *N. pleskei* VS. *P. weiningensis* | 0.998 | 1.000 | 1.000 | 1.000 |
| *N. pleskei* VS. *A. shapingensis* | ＜0.001*** | ＜0.001*** | 0.005** | 0.004** |
| *N. pleskei* VS. *O. margaretae* | 0.080 | 0.083 | 0.403 | 0.414 |
| *N. pleskei* VS. *H. gongshanensis* | 1.000 | 1.000 | 1.000 | 1.000 |
| *N. pleskei* VS. *B.gargarizans* | ＜0.001*** | ＜0.001*** | ＜0.001*** | ＜0.001*** |
| *N. pleskei* VS. *P. nigromaculatus* | 0.001** | ＜0.001*** | 0.168 | 0.152 |
| *N. pleskei* VS. *S. mammatus* | ＜0.001*** | ＜0.001*** | ＜0.001*** | ＜0.001*** |
| *N. pleskei* VS. *O. pingii* | 1.000 | 1.000 | 1.000 | 0.790 |
| *N. pleskei* VS. *R. dugritei* | 0.891 | 0.889 | 0.999 | 0.999 |
| *P. weiningensis* VS. *A. shapingensis* | ＜0.001*** | ＜0.001*** | 0.003** | 0.004** |
| *P. weiningensis* VS. *O. margaretae* | 0.041* | **0.052** | 0.375 | 0.418 |
| *P. weiningensis* VS. *H. gongshanensis* | 0.995 | 0.999 | 1.000 | 1.000 |
| *P. weiningensis* VS. *B.gargarizans* | ＜0.001*** | ＜0.001*** | ＜0.001*** | ＜0.001*** |
| *P. weiningensis* VS. *P. nigromaculatus* | ＜0.001*** | ＜0.001*** | 0.157 | 0.152 |
| *P. weiningensis* VS. *S. mammatus* | ＜0.001*** | ＜0.001*** | ＜0.001*** | ＜0.001*** |
| *P. weiningensis* VS. *O. pingii* | 0.718 | 0.896 | 0.970 | 0.998 |
| *P. weiningensis* VS. *R. dugritei* | 0.464 | 0.585 | 0.996 | 0.999 |
| *A. shapingensis* VS. *O. margaretae* | 1.000 | 1.000 | 0.987 | 0.967 |
| *A. shapingensis* VS. *H. gongshanensis* | 0.001** | ＜0.001*** | 0.010* | 0.006** |
| *A. shapingensis* VS. *B.gargarizans* | 0.998 | 0.996 | 0.788 | 0.447 |
| *A. shapingensis* VS. *P. nigromaculatus* | 0.378 | 0.599 | 0.660 | 0.628 |
| *A. shapingensis* VS. *S. mammatus* | 0.046* | **0.138** | 0.003** | 0.003** |
| *A. shapingensis* VS. *O. pingii* | ＜0.001*** | ＜0.001*** | 0.007** | 0.007** |
| *A. shapingensis* VS. *R. dugritei* | 0.836 | 0.866 | 0.994 | 1.000 |
| *O. margaretae* VS. *H. gongshanensis* | 0.099 | 0.107 | 0.440 | 0.442 |
| *O. margaretae* VS. *B.gargarizans* | 1.000 | 1.000 | 1.000 | 1.000 |
| *O. margaretae* VS. *P. nigromaculatus* | 1.000 | 1.000 | 1.000 | 1.000 |
| *O. margaretae* V S. *S. mammatus* | 1.000 | 1.000 | 1.000 | 1.000 |
| *O. margaretae* VS. *O. pingii* | 0.102 | 0.117 | 0.420 | 0.451 |
| *O. margaretae* VS. *R. dugritei* | 0.508 | 0.529 | 0.719 | 0.722 |
| *H. gongshanensis* VS. *B.gargarizans* | ＜0.001*** | ＜0.001*** | ＜0.001*** | ＜0.001*** |
| *H. gongshanensis* VS. *P. nigromaculatus* | ＜0.001*** | ＜0.001*** | 0.166 | 0.153 |
| *H. gongshanensis* VS. *S. mammatus* | ＜0.001*** | ＜0.001*** | ＜0.001*** | ＜0.001*** |
| *H. gongshanensis* VS. *O. pingii* | 1.000 | 1.000 | 1.000 | 1.000 |
| *H. gongshanensis* VS. *R. dugritei* | 0.991 | 0.985 | 1.000 | 1.000 |
| *B.gargarizans* VS. *P. nigromaculatus* | 0.999 | 1.000 | 1.000 | 1.000 |
| *B.gargarizans* VS. *S. mammatus* | 0.121 | 0.764 | 0.840 | 0.981 |
| *B.gargarizans* VS. *O. pingii* | ＜0.001*** | ＜0.001*** | ＜0.001*** | ＜0.001*** |
| *B.gargarizans* VS. *R. dugritei* | 0.149 | 0.208 | 0.048 | 0.070 |
| *P. nigromaculatus* VS. *S. mammatus* | 1.000 | 1.000 | 1.000 | 1.000 |
| *P. nigromaculatus* VS. *O. pingii* | 0.001** | 0.001** | 0.175 | 0.167 |
| *P. nigromaculatus* VS. *R. dugritei* | 0.034* | **0.065** | 0.180 | 0.176 |
| *S. mammatus* VS. *O. pingii* | ＜0.001*** | ＜0.001*** | ＜0.001*** | ＜0.001*** |
| *S. mammatus* VS. *R. dugritei* | 0.054 | 0.109 | 0.002** | 0.005** |
| *O. pingii* VS. *R. dugritei* | 0.976 | 0.988 | 1.000 | 1.000 |

*** = *P* ＜0.001; ** = *P* ＜0.01; * = *P* ＜0.05. Magnitude of the interspecific differences were reduced in bold. Red indicates the magnitude of the interspecific differences were increased.
