# Supplementary material for: Effects of long-term preservation on amphibian body conditions: implications for historical morphological research
Source: PeerJ. 2017 Sep 15;5:e3805. doi: 10.7717/peerj.3805 (PMC5602676; doi:10.7717/peerj.3805)
Supplement: Data S1 [file peerj-05-3805-s005.docx]

Data S1. Amphibian specimens and measurements used in the study.

| Species | Sex | CIB ID | Sample ID | Body length (mm)  (2006) | Body Mass  (g)  (2006) | Body length (mm)  (2015) | Body Mass (g)  (2015) | Locality |
| --- | --- | --- | --- | --- | --- | --- | --- | --- |
| *Amolops loloensis* | ♀ | CIB093644 | IOZCAS3537 | 61.0 | 20.0 | 60.6 | 15.5 | Hanyuan,Sichuan |
| *Amolops loloensis* | ♀ | CIB093645 | IOZCAS3538 | 72.7 | 34.0 | 64.5 | 19.1 | Hanyuan,Sichuan |
| *Amolops loloensis* | ♂ | CIB093646 | IOZCAS3539 | 76.1 | 40.0 | 74.2 | 31.1 | Zhaojue, Sichuan |
| *Amolops loloensis* | ♂ | CIB093647 | IOZCAS3716 | 51.9 | 14.5 | 50.5 | 11.5 | Zhaojue, Sichuan |
| *Amolops loloensis* | ♂ | CIB093648 | IOZCAS3717 | 56.1 | 17.0 | 55.4 | 13.9 | Zhaojue, Sichuan |
| *Amolops loloensis* | ♂ | CIB093649 | IOZCAS3718 | 55.0 | 16.5 | 53.0 | 12.9 | Zhaojue, Sichuan |
| *Amolops loloensis* | ♀ | CIB093650 | IOZCAS3719 | 69.9 | 34.0 | 68.6 | 28.7 | Zhaojue, Sichuan |
| *Amolops loloensis* | ♀ | CIB093651 | IOZCAS3720 | 75.8 | 41.0 | 73.6 | 35.0 | Zhaojue, Sichuan |
| *Amolops loloensis* | ♀ | CIB093652 | IOZCAS3721 | 71.5 | 35.5 | 68.9 | 29.6 | Zhaojue, Sichuan |
| *Amolops loloensis* | ♀ | CIB093653 | IOZCAS3722 | 58.6 | 20.0 | 58.4 | 16.1 | Zhaojue, Sichuan |
| *Amolops loloensis* | ♀ | CIB093654 | IOZCAS3723 | 64.3 | 27.5 | 64.0 | 22.5 | Zhaojue, Sichuan |
| *Amolops loloensis* | ♀ | CIB093655 | IOZCAS3724 | 70.3 | 33.5 | 68.4 | 27.9 | Zhaojue, Sichuan |
| *Amolops loloensis* | J | CIB093656 | IOZCAS3725 | 50.3 | 13.0 | 49.1 | 9.8 | Zhaojue, Sichuan |
| *Amolops loloensis* | ♀ | CIB093657 | IOZCAS3726 | 69.4 | 33.0 | 68.4 | 26.9 | Zhaojue, Sichuan |
| *Amolops loloensis* | ♀ | CIB093658 | IOZCAS3728 | 74.8 | 34.5 | 70.6 | 29.3 | Zhaojue, Sichuan |
| *Amolops loloensis* | ♂ | CIB093659 | IOZCAS3731 | 60.4 | 19.5 | 57.2 | 15.8 | Zhaojue, Sichuan |
| *Amolops loloensis* | J | CIB093673 | IOZCAS3732 | 55.9 | 15.0 | 49.8 | 11.6 | Zhaojue, Sichuan |
| *Amolops loloensis* | J | CIB093660 | IOZCAS3733 | 51.4 | 10.5 | 48.6 | 8.0 | Zhaojue, Sichuan |
| *Amolops loloensis* | J | CIB093661 | IOZCAS3734 | 44.9 | 7.5 | 41.1 | 5.6 | Zhaojue, Sichuan |
| *Amolops loloensis* | J | CIB093662 | IOZCAS3735 | 40.9 | 6.5 | 39.2 | 4.5 | Zhaojue, Sichuan |
| *Amolops loloensis* | J | CIB093663 | IOZCAS3736 | 38.5 | 5.0 | 36.3 | 3.5 | Zhaojue, Sichuan |
| *Amolops loloensis* | ♂ | CIB093664 | IOZCAS3808 | 55.1 | 15.0 | 50.9 | 11.9 | Zhaojue, Sichuan |
| *Amolops loloensis* | ♀ | CIB093665 | IOZCAS3809 | 56.4 | 14.0 | 52.9 | 10.2 | Zhaojue, Sichuan |
| *Amolops loloensis* | J | CIB093666 | IOZCAS3810 | 44.9 | 7.0 | 43.2 | 6.0 | Zhaojue, Sichuan |
| *Amolops loloensis* | J | CIB093667 | IOZCAS3811 | 37.2 | 4.5 | 36.1 | 3.7 | Zhaojue, Sichuan |
| *Amolops loloensis* | J | CIB093668 | IOZCAS3812 | 33.9 | 3.5 | 32.0 | 2.4 | Zhaojue, Sichuan |
| *Fejervarya multistriata* | ♀ | CIB093636 | IOZCAS3744 | 41.3 | 6.0 | 39.3 | 4.1 | Xichang, Sichuan |
| *Fejervarya multistriata* | ♂ | CIB093490 | IOZCAS3746 | 38.7 | 5.0 | 35.8 | 3.5 | Xichang, Sichuan |
| *Fejervarya multistriata* | ♂ | CIB093637 | IOZCAS3748 | 37.1 | 4.0 | 32.6 | 2.6 | Xichang, Sichuan |
| *Fejervarya multistriata* | ♂ | CIB093638 | IOZCAS3749 | 37.6 | 4.5 | 35.0 | 3.2 | Xichang, Sichuan |
| *Fejervarya multistriata* | ♂ | CIB093491 | IOZCAS3750 | 38.6 | 5.0 | 35.5 | 3.6 | Xichang, Sichuan |
| *Scutiger glandulatus* | ♂ | CIB094676 | IOZCAS3978 | 68.1 | 30.5 | 64.5 | 24.2 | Kangding, Sichuan |
| *Scutiger glandulatus* | ♂ | CIB094677 | IOZCAS3979 | 71.0 | 34.0 | 67.6 | 27.3 | Kangding, Sichuan |
| *Scutiger glandulatus* | ♂ | CIB094678 | IOZCAS3980 | 74.1 | 36.0 | 68.4 | 31.1 | Kangding, Sichuan |
| *Scutiger glandulatus* | ♂ | CIB094679 | IOZCAS3981 | 76.4 | 40.1 | 70.3 | 33.2 | Kangding, Sichuan |
| *Scutiger glandulatus* | ♂ | CIB094680 | IOZCAS3982 | 74.2 | 35.0 | 67.4 | 27.3 | Kangding, Sichuan |
| *Scutiger glandulatus* | ♂ | CIB094725 | IOZCAS3983 | 78.3 | 38.0 | 71.6 | 30.1 | Kangding, Sichuan |
| *Scutiger glandulatus* | ♂ | CIB094681 | IOZCAS3984 | 62.4 | 20.5 | 57.9 | 16.1 | Kangding, Sichuan |
| *Scutiger glandulatus* | ♂ | CIB094682 | IOZCAS3985 | 60.9 | 19.0 | 57.3 | 15.0 | Kangding, Sichuan |
| *Scutiger glandulatus* | ♀ | CIB093607 | IOZCAS3986 | 60.1 | 19.0 | 56.4 | 15.0 | Kangding, Sichuan |
| *Scutiger glandulatus* | ♂ | CIB094683 | IOZCAS3988 | 76.5 | 44.0 | 68.4 | 36.4 | Kangding, Sichuan |
| *Scutiger glandulatus* | ♂ | CIB094684 | IOZCAS3989 | 76.4 | 40.0 | 72.6 | 30.3 | Kangding, Sichuan |
| *Scutiger glandulatus* | ♂ | CIB094685 | IOZCAS3990 | 75.9 | 38.0 | 71.4 | 31.0 | Kangding, Sichuan |
| *Scutiger glandulatus* | ♂ | CIB094686 | IOZCAS3991 | 76.0 | 38.5 | 70.4 | 30.5 | Kangding, Sichuan |
| *Scutiger glandulatus* | ♀ | CIB094726 | IOZCAS3992 | 77.3 | 34.0 | 75.2 | 25.0 | Kangding, Sichuan |
| *Scutiger glandulatus* | ♀ | CIB093608 | IOZCAS3993 | 51.7 | 10.0 | 47.3 | 9.0 | Kangding, Sichuan |
| *Nanorana pleskei* | ♂ | CIB093492 | IOZCAS3862 | 31.3 | 5.0 | 30.0 | 1.6 | Jiulong, Sichuan |
| *Nanorana pleskei* | ♂ | CIB093493 | IOZCAS3863 | 37.3 | 4.0 | 35.0 | 2.4 | Jiulong, Sichuan |
| *Nanorana pleskei* | ♂ | CIB093494 | IOZCAS3864 | 35.3 | 4.5 | 33.9 | 2.1 | Jiulong, Sichuan |
| *Nanorana pleskei* | ♂ | CIB093495 | IOZCAS3865 | 35.1 | 3.5 | 32.2 | 1.8 | Jiulong, Sichuan |
| *Nanorana pleskei* | ♂ | CIB093496 | IOZCAS3866 | 34.6 | 3.0 | 31.1 | 1.8 | Jiulong, Sichuan |
| *Nanorana pleskei* | ♂ | CIB093497 | IOZCAS3868 | 37.7 | 4.5 | 34.3 | 2.2 | Jiulong, Sichuan |
| *Nanorana pleskei* | ♀ | CIB093498 | IOZCAS3870 | 33.5 | 3.0 | 30.9 | 1.5 | Jiulong, Sichuan |
| *Nanorana pleskei* | J | CIB093499 | IOZCAS3871 | 26.8 | 1.5 | 23.5 | 1.0 | Jiulong, Sichuan |
| *Nanorana pleskei* | J | CIB093500 | IOZCAS3872 | 27.8 | 2.0 | 24.8 | 1.0 | Jiulong, Sichuan |
| *Nanorana pleskei* | J | CIB093501 | IOZCAS3873 | 21.2 | 1.0 | 16.7 | 0.5 | Jiulong, Sichuan |
| *Pseudorana weiningensis* | ♂ | CIB093596 | IOZCAS3761 | 33.0 | 3.5 | 31.4 | 2.5 | Zhaojue, Sichuan |
| *Pseudorana weiningensis* | ♂ | CIB093597 | IOZCAS3762 | 33.5 | 4.0 | 31.8 | 2.9 | Zhaojue, Sichuan |
| *Pseudorana weiningensis* | ♂ | CIB093598 | IOZCAS3763 | 33.5 | 3.5 | 32.2 | 2.8 | Zhaojue, Sichuan |
| *Pseudorana weiningensis* | J | CIB093599 | IOZCAS3765 | 23.4 | 1.5 | 22.1 | 1.1 | Zhaojue, Sichuan |
| *Pseudorana weiningensis* | J | CIB093600 | IOZCAS3766 | 23.9 | 1.5 | 23.8 | 1.2 | Zhaojue, Sichuan |
| *Pseudorana weiningensis* | J | CIB093601 | IOZCAS3767 | 22.2 | 1.5 | 21.2 | 1.1 | Zhaojue, Sichuan |
| *Pseudorana weiningensis* | J | CIB093602 | IOZCAS3768 | 24.4 | 1.5 | 21.3 | 1.2 | Zhaojue, Sichuan |
| *Pseudorana weiningensis* | J | CIB094486 | IOZCAS3769 | 23.5 | 1.5 | 21.6 | 1.0 | Zhaojue, Sichuan |
| *Atympanophrys shapingensis* | ♀ | CIB093464 | IOZCAS3698 | 81.6 | 52.0 | 80.0 | 41.5 | Zhaojue, Sichuan |
| *Atympanophrys shapingensis* | J | CIB093465 | IOZCAS3699 | 54.2 | 15.0 | 53.2 | 11.7 | Zhaojue, Sichuan |
| *Atympanophrys shapingensis* | J | CIB093466 | IOZCAS3700 | 34.7 | 4.5 | 34.1 | 3.1 | Zhaojue, Sichuan |
| *Atympanophrys shapingensis* | ♀ | CIB093467 | IOZCAS3705 | 79.3 | 35.5 | 70.0 | 27.2 | Zhaojue, Sichuan |
| *Atympanophrys shapingensis* | ♂ | CIB093468 | IOZCAS3706 | 75.5 | 34.5 | 72.5 | 25.6 | Zhaojue, Sichuan |
| *Atympanophrys shapingensis* | ♂ | CIB093593 | IOZCAS3708 | 81.5 | 42.0 | 76.0 | 29.0 | Zhaojue, Sichuan |
| *Atympanophrys shapingensis* | ♂ | CIB093469 | IOZCAS3709 | 65.5 | 23.0 | 63.2 | 16.7 | Zhaojue, Sichuan |
| *Atympanophrys shapingensis* | ♀ | CIB093470 | IOZCAS3710 | 79.6 | 31.0 | 77.0 | 24.2 | Zhaojue, Sichuan |
| *Atympanophrys shapingensis* | J | CIB093471 | IOZCAS3712 | 48.9 | 9.0 | 47.2 | 6.7 | Zhaojue, Sichuan |
| *Atympanophrys shapingensis* | ♀ | CIB093472 | IOZCAS3801 | 81.9 | 42.5 | 75.2 | 32.2 | Yuexi,Sichuan |
| *Atympanophrys shapingensis* | ♀ | CIB093473 | IOZCAS3802 | 58.4 | 14.5 | 54.5 | 10.7 | Yuexi,Sichuan |
| *Atympanophrys shapingensis* | ♂ | CIB093474 | IOZCAS3803 | 59.3 | 16.0 | 57.7 | 12.4 | Yuexi,Sichuan |
| *Atympanophrys shapingensis* | J | CIB093475 | IOZCAS3804 | 50.4 | 11.5 | 49.6 | 6.8 | Yuexi,Sichuan |
| *Atympanophrys shapingensis* | J | CIB093476 | IOZCAS3805 | 45.6 | 7.5 | 42.8 | 5.5 | Yuexi,Sichuan |
| *Atympanophrys shapingensis* | J | CIB093477 | IOZCAS3806 | 39.1 | 5.0 | 36.9 | 3.4 | Yuexi,Sichuan |
| *Atympanophrys shapingensis* | J | CIB093478 | IOZCAS3807 | 32.8 | 3.0 | 30.5 | 1.9 | Yuexi,Sichuan |
| *Atympanophrys shapingensis* | ♀ | CIB093479 | IOZCAS3860 | 61.1 | 23.0 | 56.9 | 19.4 | Yuexi,Sichuan |
| *Atympanophrys shapingensis* | ♀ | CIB093480 | IOZCAS3861 | 67.0 | 23.5 | 63.2 | 18.8 | Yuexi,Sichuan |
| *Odorrana margaretae* | ♀ | CIB093450 | IOZCAS3501 | 108.8 | 89.0 | 105.2 | 73.3 | Dujiangyan, Sichuan |
| *Odorrana margaretae* | ♀ | CIB093451 | IOZCAS3502 | 102.8 | 92.0 | 100.8 | 86.5 | Dujiangyan, Sichuan |
| *Odorrana margaretae* | ♀ | CIB093452 | IOZCAS3503 | 92.3 | 69.0 | 91.2 | 68.3 | Dujiangyan, Sichuan |
| *Odorrana margaretae* | ♀ | CIB093453 | IOZCAS3504 | 103.0 | 96.0 | 100.9 | 78.6 | Dujiangyan, Sichuan |
| *Odorrana margaretae* | ♀ | CIB093454 | IOZCAS3505 | 101.2 | 94.0 | 100.6 | 86.2 | Dujiangyan, Sichuan |
| *Odorrana margaretae* | J | CIB093455 | IOZCAS3693 | 55.4 | 13.0 | 48.7 | 9.6 | Miyi, Sichuan |
| *Odorrana margaretae* | J | CIB093456 | IOZCAS3694 | 60.1 | 18.5 | 56.1 | 13.8 | Miyi, Sichuan |
| *Odorrana margaretae* | J | CIB093457 | IOZCAS3695 | 52.4 | 13.5 | 52.4 | 10.3 | Miyi, Sichuan |
| *Odorrana margaretae* | J | CIB093458 | IOZCAS3696 | 35.2 | 3.5 | 33.2 | 2.7 | Miyi, Sichuan |
| *Hyla gongshanensis* | ♂ | CIB093615 | IOZCAS3751 | 35.8 | 4.0 | 33.2 | 2.2 | Xichang, Sichuan |
| *Hyla gongshanensis* | ♂ | CIB093616 | IOZCAS3753 | 29.7 | 3.0 | 28.2 | 1.5 | Xichang, Sichuan |
| *Hyla gongshanensis* | ♂ | CIB093617 | IOZCAS3754 | 29.4 | 3.0 | 27.6 | 1.7 | Xichang, Sichuan |
| *Hyla gongshanensis* | ♂ | CIB093618 | IOZCAS3755 | 28.7 | 2.5 | 25.6 | 1.3 | Xichang, Sichuan |
| *Hyla gongshanensis* | ♂ | CIB093621 | IOZCAS3825 | 36.0 | 4.5 | 32.8 | 2.3 | Yuexi,Sichuan |
| *Hyla gongshanensis* | ♀ | CIB093622 | IOZCAS3826 | 45.9 | 9.0 | 40.9 | 5.2 | Yuexi,Sichuan |
| *Bufo gargarizans* | ♀ | CIB093550 | IOZCAS3598 | 85.0 | 63.0 | 77.0 | 41.7 | Mianning, Sichuan |
| *Bufo gargarizans* | ♀ | CIB093552 | IOZCAS3600 | 83.0 | 58.0 | 77.4 | 52.6 | Mianning, Sichuan |
| *Bufo gargarizans* | ♀ | CIB093543 | IOZCAS3507 | 68.5 | 31.0 | 64.7 | 25.3 | Pengzhou,Sichuan |
| *Bufo gargarizans* | ♀ | CIB093544 | IOZCAS3508 | 62.6 | 21.0 | 60.2 | 20.5 | Pengzhou,Sichuan |
| *Bufo gargarizans* | ♂ | CIB093547 | IOZCAS3587 | 60.8 | 29.0 | 60.6 | 20.4 | Mianning, Sichuan |
| *Bufo gargarizans* | ♂ | CIB093548 | IOZCAS3588 | 65.8 | 29.0 | 64.2 | 22.3 | Mianning, Sichuan |
| *Bufo gargarizans* | ♂ | CIB093549 | IOZCAS3597 | 58.9 | 19.0 | 56.0 | 14.0 | Mianning, Sichuan |
| *Bufo gargarizans* | ♀ | CIB093551 | IOZCAS3599 | 78.0 | 38.0 | 74.7 | 31.7 | Mianning, Sichuan |
| *Bufo gargarizans* | ♀ | CIB093553 | IOZCAS3601 | 74.0 | 37.0 | 69.5 | 30.1 | Mianning, Sichuan |
| *Bufo gargarizans* | ♀ | CIB093545 | IOZCAS3651 | 82.8 | 53.0 | 76.8 | 44.9 | Yanyuan,Sichuan |
| *Bufo gargarizans* | J | CIB093546 | IOZCAS3654 | 51.3 | 14.0 | 50.8 | 10.9 | Muli,Sichuan |
| *Bufo gargarizans* | J | CIB093555 | IOZCAS3737 | 60.9 | 21.5 | 55.9 | 16.0 | Zhaojue, Sichuan |
| *Bufo gargarizans* | J | CIB093556 | IOZCAS3738 | 44.5 | 8.0 | 39.2 | 5.9 | Zhaojue, Sichuan |
| *Bufo gargarizans* | ♂ | CIB093557 | IOZCAS3739 | 65.4 | 30.0 | 62.0 | 24.2 | Zhaojue, Sichuan |
| *Bufo gargarizans* | ♀ | CIB093558 | IOZCAS3740 | 73.9 | 37.0 | 71.4 | 29.6 | Zhaojue, Sichuan |
| *Bufo gargarizans* | ♀ | CIB093559 | IOZCAS3741 | 79.9 | 55.0 | 77.1 | 44.0 | Zhaojue, Sichuan |
| *Bufo gargarizans* | ♀ | CIB093560 | IOZCAS3742 | 95.4 | 84.0 | 89.1 | 61.4 | Xichang, Sichuan |
| *Bufo gargarizans* | ♀ | CIB093561 | IOZCAS3774 | 75.5 | 36.0 | 71.6 | 30.0 | Zhaojue, Sichuan |
| *Bufo gargarizans* | ♂ | CIB093562 | IOZCAS3777 | 64.6 | 21.5 | 60.2 | 16.6 | Zhaojue, Sichuan |
| *Bufo gargarizans* | ♂ | CIB093563 | IOZCAS3818 | 64.8 | 25.0 | 60.6 | 22.4 | Yuexi,Sichuan |
| *Bufo gargarizans* | ♂ | CIB093564 | IOZCAS3819 | 62.4 | 22.5 | 58.6 | 17.6 | Yuexi,Sichuan |
| *Bufo gargarizans* | ♀ | CIB093565 | IOZCAS3820 | 73.4 | 39.0 | 71.6 | 35.0 | Yuexi,Sichuan |
| *Bufo gargarizans* | J | CIB093566 | IOZCAS3821 | 54.4 | 16.5 | 52.1 | 13.6 | Yuexi,Sichuan |
| *Bufo gargarizans* | ♀ | CIB093575 | IOZCAS3874 | 80.9 | 51.0 | 77.1 | 40.4 | Jiulong, Sichuan |
| *Bufo gargarizans* | J | CIB093576 | IOZCAS3875 | 44.5 | 9.0 | 42.9 | 7.5 | Jiulong, Sichuan |
| *Bufo gargarizans* | ♀ | CIB093567 | IOZCAS3939 | 79.3 | 40.0 | 73.5 | 34.0 | Jiulong, Sichuan |
| *Bufo gargarizans* | ♂ | CIB093568 | IOZCAS3960 | 72.1 | 27.0 | 69.5 | 21.6 | Jiulong, Sichuan |
| *Bufo gargarizans* | ♂ | CIB093569 | IOZCAS3961 | 65.1 | 22.0 | 62.9 | 17.2 | Jiulong, Sichuan |
| *Bufo gargarizans* | ♂ | CIB093570 | IOZCAS3966 | 70.0 | 27.5 | 66.8 | 21.5 | Jiulong, Sichuan |
| *Bufo gargarizans* | ♂ | CIB093571 | IOZCAS3967 | 66.2 | 19.0 | 63.9 | 14.8 | Jiulong, Sichuan |
| *Bufo gargarizans* | ♀ | CIB093572 | IOZCAS3968 | 63.2 | 19.0 | 61.8 | 18.8 | Jiulong, Sichuan |
| *Bufo gargarizans* | ♀ | CIB093573 | IOZCAS3969 | 81.1 | 40.5 | 77.4 | 32.3 | Jiulong, Sichuan |
| *Bufo gargarizans* | ♀ | CIB093574 | IOZCAS3970 | 85.0 | 54.0 | 80.5 | 41.5 | Jiulong, Sichuan |
| *Pelophylax nigromaculatus* | ♀ | CIB093640 | IOZCAS3671 | 76.1 | 47.0 | 73.0 | 39.4 | Miyi, Sichuan |
| *Pelophylax nigromaculatus* | ♂ | CIB093641 | IOZCAS3672 | 80.3 | 41.0 | 71.1 | 29.5 | Miyi, Sichuan |
| *Pelophylax nigromaculatus* | ♂ | CIB093642 | IOZCAS3673 | 83.5 | 59.0 | 78.9 | 43.6 | Miyi, Sichuan |
| *Pelophylax nigromaculatus* | ♀ | CIB093643 | IOZCAS3675 | 79.6 | 52.0 | 73.3 | 39.2 | Miyi, Sichuan |
| *Pelophylax nigromaculatus* | ♂ | CIB093639 | IOZCAS3743 | 65.5 | 24.5 | 60.9 | 19.0 | Xichang, Sichuan |
| *Scutiger mammatus* | ♀ | CIB094709 | IOZCAS3904 | 84.2 | 52.0 | 77.1 | 41.5 | Jiulong, Sichuan |
| *Scutiger mammatus* | ♀ | CIB094700 | IOZCAS3890 | 86.4 | 51.0 | 79.3 | 41.6 | Jiulong, Sichuan |
| *Scutiger mammatus* | ♂ | CIB094701 | IOZCAS3896 | 78.2 | 56.0 | 71.1 | 41.1 | Jiulong, Sichuan |
| *Scutiger mammatus* | ♂ | CIB094702 | IOZCAS3897 | 73.9 | 40.0 | 68.2 | 30.2 | Jiulong, Sichuan |
| *Scutiger mammatus* | ♂ | CIB094703 | IOZCAS3898 | 80.4 | 51.0 | 74.7 | 44.4 | Jiulong, Sichuan |
| *Scutiger mammatus* | ♂ | CIB094704 | IOZCAS3899 | 78.0 | 42.0 | 73.1 | 31.8 | Jiulong, Sichuan |
| *Scutiger mammatus* | ♂ | CIB094705 | IOZCAS3900 | 77.2 | 46.0 | 71.4 | 32.9 | Jiulong, Sichuan |
| *Scutiger mammatus* | ♂ | CIB094706 | IOZCAS3901 | 73.5 | 37.0 | 64.3 | 26.6 | Jiulong, Sichuan |
| *Scutiger mammatus* | ♀ | CIB094707 | IOZCAS3902 | 68.6 | 21.5 | 66.5 | 17.1 | Jiulong, Sichuan |
| *Scutiger mammatus* | ♀ | CIB094708 | IOZCAS3903 | 76.7 | 29.0 | 72.5 | 23.0 | Jiulong, Sichuan |
| *Scutiger mammatus* | ♂ | CIB094712 | IOZCAS3922 | 86.8 | 67.0 | 78.5 | 45.5 | Jiulong, Sichuan |
| *Scutiger mammatus* | ♂ | CIB094713 | IOZCAS3923 | 79.2 | 49.0 | 71.7 | 35.6 | Jiulong, Sichuan |
| *Scutiger mammatus* | ♂ | CIB094714 | IOZCAS3924 | 73.9 | 40.0 | 72.1 | 27.9 | Jiulong, Sichuan |
| *Scutiger mammatus* | ♀ | CIB094715 | IOZCAS3925 | 77.1 | 35.0 | 72.8 | 26.7 | Jiulong, Sichuan |
| *Scutiger mammatus* | ♀ | CIB094716 | IOZCAS3926 | 74.7 | 36.0 | 66.9 | 27.5 | Jiulong, Sichuan |
| *Scutiger mammatus* | ♂ | CIB094717 | IOZCAS3949 | 76.4 | 40.0 | 69.8 | 31.6 | Jiulong, Sichuan |
| *Scutiger mammatus* | ♂ | CIB094718 | IOZCAS3950 | 77.4 | 43.0 | 68.9 | 30.9 | Jiulong, Sichuan |
| *Scutiger mammatus* | ♂ | CIB094719 | IOZCAS3951 | 68.9 | 25.0 | 63.6 | 20.4 | Jiulong, Sichuan |
| *Scutiger mammatus* | ♂ | CIB094720 | IOZCAS3952 | 77.9 | 38.0 | 73.0 | 31.5 | Jiulong, Sichuan |
| *Scutiger mammatus* | ♂ | CIB094721 | IOZCAS3953 | 63.5 | 21.0 | 58.9 | 16.7 | Jiulong, Sichuan |
| *Scutiger mammatus* | ♂ | CIB094722 | IOZCAS3954 | 64.2 | 23.0 | 59.2 | 16.1 | Jiulong, Sichuan |
| *Scutiger mammatus* | ♀ | CIB094723 | IOZCAS3955 | 71.4 | 26.5 | 63.3 | 20.9 | Jiulong, Sichuan |
| *Scutiger mammatus* | ♀ | CIB094724 | IOZCAS3956 | 72.1 | 29.0 | 64.7 | 23.2 | Jiulong, Sichuan |
| *Scutiger mammatus* | ♂ | CIB094710 | IOZCAS4007 | 85.5 | 60.0 | 79.2 | 43.3 | Jiulong, Sichuan |
| *Scutiger mammatus* | ♂ | CIB094711 | IOZCAS4008 | 86.7 | 60.0 | 77.9 | 47.0 | Jiulong, Sichuan |
| *Scutiger mammatus* | ♂ | CIB094687 | IOZCAS4010 | 82.1 | 44.5 | 78.6 | 38.3 | Jiulong, Sichuan |
| *Scutiger mammatus* | ♀ | CIB094698 | IOZCAS3637 | 89.9 | 58.0 | 83.7 | 51.1 | Yanyuan,Sichuan |
| *Scutiger mammatus* | ♂ | CIB094699 | IOZCAS3639 | 100.0 | 95.0 | 91.2 | 75.8 | Yanyuan,Sichuan |
| *Oreolalax pingii* | J | CIB093593 | IOZCAS3708 | 39.8 | 6.0 | 36.4 | 3.1 | Zhaojue, Sichuan |
| *Oreolalax pingii* | J | CIB093594 | IOZCAS3772 | 29.9 | 2.0 | 27.3 | 1.7 | Zhaojue, Sichuan |
| *Oreolalax pingii* | J | CIB093595 | IOZCAS3773 | 27.5 | 2.0 | 25.7 | 1.5 | Zhaojue, Sichuan |
| *Oreolalax pingii* | ♂ | CIB094474 | IOZCAS3779 | 39.4 | 5.0 | 38.1 | 3.2 | Zhaojue, Sichuan |
| *Oreolalax pingii* | ♂ | CIB094475 | IOZCAS3780 | 40.7 | 5.0 | 38.7 | 3.6 | Zhaojue, Sichuan |
| *Oreolalax pingii* | ♂ | CIB094476 | IOZCAS3781 | 45.9 | 7.5 | 42.1 | 5.7 | Zhaojue, Sichuan |
| *Oreolalax pingii* | ♂ | CIB094477 | IOZCAS3783 | 41.1 | 6.0 | 40.2 | 4.2 | Zhaojue, Sichuan |
| *Oreolalax pingii* | J | CIB094729 | IOZCAS3784 | 31.2 | 3.0 | 30.0 | 2.3 | Zhaojue, Sichuan |
| *Oreolalax pingii* | J | CIB094478 | IOZCAS3785 | 29.2 | 2.5 | 27.1 | 1.8 | Zhaojue, Sichuan |
| *Oreolalax pingii* | J | CIB094479 | IOZCAS3786 | 29.2 | 2.5 | 26.6 | 1.7 | Zhaojue, Sichuan |
| *Oreolalax pingii* | J | CIB094480 | IOZCAS3787 | 30.5 | 3.0 | 28.6 | 1.9 | Zhaojue, Sichuan |
| *Oreolalax pingii* | J | CIB094481 | IOZCAS3788 | 33.1 | 3.0 | 31.4 | 2.3 | Zhaojue, Sichuan |
| *Oreolalax pingii* | J | CIB094482 | IOZCAS3789 | 30.8 | 2.5 | 26.7 | 1.8 | Zhaojue, Sichuan |
| *Oreolalax pingii* | J | CIB094483 | IOZCAS3790 | 26.4 | 2.0 | 25.0 | 1.4 | Zhaojue, Sichuan |
| *Rhacophorus dugritei* | ♂ | CIB093528 | IOZCAS3580 | 43.5 | 9.0 | 40.8 | 6.4 | Mianning, Sichuan |
| *Rhacophorus dugritei* | ♂ | CIB093529 | IOZCAS3582 | 39.0 | 6.0 | 36.9 | 4.9 | Mianning, Sichuan |
| *Rhacophorus dugritei* | ♂ | CIB093530 | IOZCAS3583 | 41.9 | 7.0 | 38.2 | 6.1 | Mianning, Sichuan |
| *Rhacophorus dugritei* | ♀ | CIB093531 | IOZCAS3584 | 61.8 | 25.0 | 59.4 | 20.6 | Mianning, Sichuan |
| *Rhacophorus dugritei* | ♂ | CIB093532 | IOZCAS3585 | 42.5 | 10.0 | 40.3 | 6.2 | Mianning, Sichuan |
