# Supplementary material for: Effects of long-term preservation on amphibian body conditions: implications for historical morphological research
Source: PeerJ. 2017 Sep 15;5:e3805. doi: 10.7717/peerj.3805 (PMC5602676; doi:10.7717/peerj.3805)
Supplement: Data S2 [file peerj-05-3805-s006.docx]

Data S2. Original data for test of inter-observer bias.

| Trait  Species | CIB ID | Sample ID | Body length (mm) | | Body mass (g) | |
| --- | --- | --- | --- | --- | --- | --- |
|  |  |  | GCS | CL | GCS | CL |
| *Pseudorana weiningensis* | CIB093596 | IOZCAS3761 | 31.4 | 31.5 | 2.5 | 2.5 |
| *Pseudorana weiningensis* | CIB093597 | IOZCAS3762 | 31.8 | 31.8 | 2.9 | 2.9 |
| *Pseudorana weiningensis* | CIB093598 | IOZCAS3763 | 32.2 | 32.2 | 2.8 | 2.8 |
| *Pseudorana weiningensis* | CIB093599 | IOZCAS3765 | 22.1 | 22.1 | 1.1 | 1.1 |
| *Pseudorana weiningensis* | CIB093600 | IOZCAS3766 | 23.8 | 23.7 | 1.2 | 1.2 |
| *Pseudorana weiningensis* | CIB093601 | IOZCAS3767 | 21.2 | 21.1 | 1.1 | 1.1 |
| *Pseudorana weiningensis* | CIB093602 | IOZCAS3768 | 21.3 | 21.0 | 1.2 | 1.1 |
| *Pseudorana weiningensis* | CIB094486 | IOZCAS3769 | 21.6 | 21.6 | 1.0 | 1.0 |
| *Amolops loloensis* | CIB093644 | IOZCAS3537 | 63.6 | 63.2 | 16.4 | 16.8 |
| *Amolops loloensis* | CIB093645 | IOZCAS3538 | 72.6 | 72.7 | 30.4 | 30.2 |
| *Amolops loloensis* | CIB093646 | IOZCAS3539 | 73.9 | 71.9 | 35.8 | 36.1 |
| *Amolops loloensis* | CIB093647 | IOZCAS3716 | 53.1 | 51.5 | 11.0 | 11.4 |
| *Amolops loloensis* | CIB093648 | IOZCAS3717 | 56.0 | 55.4 | 13.4 | 13.3 |
| *Amolops loloensis* | CIB093649 | IOZCAS3718 | 53.1 | 51.9 | 12.7 | 12.9 |
| *Amolops loloensis* | CIB093650 | IOZCAS3719 | 69.6 | 68.5 | 27.4 | 27.3 |
| *Amolops loloensis* | CIB093651 | IOZCAS3720 | 73.3 | 73.7 | 33.4 | 33.2 |
| *Amolops loloensis* | CIB093652 | IOZCAS3721 | 67.9 | 69.3 | 28.8 | 28.5 |
| *Amolops loloensis* | CIB093653 | IOZCAS3722 | 56.6 | 57.9 | 15.4 | 15.8 |
| *Amolops loloensis* | CIB093654 | IOZCAS3723 | 62.3 | 61.9 | 21.7 | 21.2 |
| *Amolops loloensis* | CIB093655 | IOZCAS3724 | 70.2 | 69.2 | 27.4 | 27.3 |
| *Amolops loloensis* | CIB093656 | IOZCAS3725 | 50.8 | 49.4 | 9.4 | 9.8 |
| *Amolops loloensis* | CIB093657 | IOZCAS3726 | 68.3 | 67.8 | 24.2 | 24.1 |
| *Amolops loloensis* | CIB093658 | IOZCAS3728 | 70.5 | 70.0 | 28.7 | 28.3 |
| *Amolops loloensis* | CIB093659 | IOZCAS3731 | 57.1 | 57.8 | 15.1 | 15.4 |
| *Amolops loloensis* | CIB093673 | IOZCAS3732 | 50.1 | 51.4 | 10.0 | 10.1 |
| *Amolops loloensis* | CIB093660 | IOZCAS3733 | 47.3 | 47.6 | 7.3 | 7.5 |
| *Amolops loloensis* | CIB093661 | IOZCAS3734 | 40.0 | 40.3 | 5.3 | 5.4 |
| *Amolops loloensis* | CIB093662 | IOZCAS3735 | 38.4 | 39.2 | 4.1 | 4.3 |
| *Amolops loloensis* | CIB093663 | IOZCAS3736 | 36.3 | 36.7 | 3.2 | 3.3 |
| *Amolops loloensis* | CIB093664 | IOZCAS3808 | 49.7 | 50.5 | 10.3 | 10.5 |
| *Amolops loloensis* | CIB093665 | IOZCAS3809 | 52.6 | 53.2 | 9.8 | 10.1 |
| *Amolops loloensis* | CIB093666 | IOZCAS3810 | 42.9 | 42.7 | 5.3 | 5.5 |
| *Amolops loloensis* | CIB093667 | IOZCAS3811 | 36.0 | 35.8 | 3.2 | 3.3 |
| *Amolops loloensis* | CIB093668 | IOZCAS3812 | 32.0 | 32.4 | 2.3 | 2.3 |
| *Nanorana pleskei* | CIB093492 | IOZCAS3862 | 29.6 | 29.7 | 1.6 | 1.6 |
| *Nanorana pleskei* | CIB093493 | IOZCAS3863 | 33.9 | 33.6 | 2.4 | 2.4 |
| *Nanorana pleskei* | CIB093494 | IOZCAS3864 | 33.6 | 33.4 | 2.1 | 2.2 |
| *Nanorana pleskei* | CIB093495 | IOZCAS3865 | 31.2 | 31.3 | 1.7 | 1.7 |
| *Nanorana pleskei* | CIB093496 | IOZCAS3866 | 29.5 | 30.0 | 1.7 | 1.7 |
| *Nanorana pleskei* | CIB093497 | IOZCAS3868 | 32.7 | 32.5 | 1.9 | 2.0 |
| *Nanorana pleskei* | CIB093498 | IOZCAS3870 | 29.9 | 30.3 | 1.5 | 1.5 |
| *Nanorana pleskei* | CIB093499 | IOZCAS3871 | 22.0 | 22.2 | 0.9 | 0.9 |
| *Nanorana pleskei* | CIB093500 | IOZCAS3872 | 24.5 | 24.6 | 0.9 | 1.0 |
| *Nanorana pleskei* | CIB093501 | IOZCAS3873 | 16.7 | 16.8 | 0.5 | 0.5 |
